# Supplementary material for: Research on the impact and mechanism of local governments’ land conveyance behavior on urban-rural integrated development—Empirical evidence from 281 prefecture-level cities in China
Source: PLoS One. 2025 Jul 7;20(7):e0327433. doi: 10.1371/journal.pone.0327433 (PMC12233302; doi:10.1371/journal.pone.0327433)
Supplement: Attachment — Submitted filename: Response to Reviewers.docx [file pone.0327433.s002.docx]

**Manuscript ID:**PONE-D-24-33248

**Title:**  Research on the Impact and Mechanism of Local Government Land Transfer Behavior on Urban Rural Integration Development——Perspectives based on land price competition and land finance dependence

**Authors name:**Long Zeng , Bin Peng' * and Jin Xie

**Response to Reviewers**

Dear Editor and Reviewers,

Thanks very much for taking your time to review this manuscript. we really appreciate all your comments and suggestions! Below, we provide a detailed response to each comment raised by the reviewers and outline the revisions made accordingly. In addition, the modifications we have made have been highlighted in red font in the re-submitted files.

**Reply to the comments of Reviewer 1**

1.Please expand on the practical and theoretical implications, and clarify the study’s significance

A：Thank you very much for your suggestions. In the introduction section, we have elaborated on the theoretical and practical significance of this study in the current research field, rather than providing a general overview. The excerpt is as follows:

*“The potential marginal contributions of this study are as follows: First, this paper is the first to systematically elucidate the impact mechanism of local governments' land transfer behavior on urban-rural integration, filling a gap in the existing literature. By constructing a unified theoretical framework, this study deeply explores the internal logic of how land transfer behavior influences urban-rural integration through channels such as labor migration, public service provision, and industrial structure upgrading. This provides a novel perspective for the theoretical discourse on the relationship between land transfer behavior and urban-rural integration.Second, this research provides theoretical support and practical guidance for local governments to optimize policies in the process of land transfer. Particularly in the context of promoting urban-rural integration, the findings of this study help local governments better understand the multidimensional impacts of land transfer behavior and thereby formulate more scientific land transfer and fiscal policies. Furthermore, the policy recommendations proposed in this study can assist governments in rationally adjusting land transfer practices to facilitate labor migration, enhance public service provision, and promote industrial structure upgrading. In doing so, this study aims to foster urban-rural integration, reduce the urban-rural gap, and achieve coordinated economic and social development.”*Please refer to lines 109 to 121 in the article for the specific details.

**Reply to the comments of Reviewer 2**

1.You have justified some of the findings. Provide references for these sentences. For example, in part 4.2.1 Regression results and analysis by region, some sentences about the possible reasons for the difference in results in different regions need to be referenced.

A：Thank you very much for your suggestions. Regarding the possible reasons for the differences in results across regions, we have further cited relevant literature in the paper to supplement the analysis of the causes of regional disparities and emphasized how these factors affect urban-rural integration, thereby enhancing the theoretical depth and practical significance of the study. The excerpt is as follows:

*“In China’s eastern and central regions, industrial development occurred relatively early, and economic development has gradually transitioned from traditional heavy industries to high-tech and service industries[53]. The relatively high labor costs in these regions have made low-priced industrial land transfers less effective in attracting enterprises, thereby diminishing their impact on urban-rural integration.”*Please refer to lines 396 to 399 in the article for the specific details.

*“From the regression results on land fiscal dependence, it is evident that the dependence on land finance has a significant negative effect on urban-rural integration in western China, while it exhibits a significant positive effect in the central region and no significant impact in the eastern region. The potential reasons for these findings include the following: the western region’s underdeveloped economy, combined with low household income and consumption levels, reduces enterprises' willingness to engage in diversified investments[54]. This situation exacerbates issues such as industrial structure rigidity and homogeneity caused by local governments' high-priced transfers of commercial and residential land, ultimately hindering urban-rural integration.”*Please refer to lines 400 to 406 in the article for the specific details.

2.In Table 6, the column of social criteria is repeated twice. Please correct.

A：Thank you very much for your thorough review. First, due to the addition of relevant tables presenting unit root and cointegration test results during the revision process, Table 6 has now been renumbered as Table 8. Second, regarding the issue of the social integration dimension being listed twice, this is due to the limitations of table length. In this paper, Table 8 presents the social integration dimension in two separate sections: the upper section displays the regression results of land price competition on urban-rural social integration, while the lower section shows the regression results of land fiscal dependence on urban-rural social integration. To avoid any misunderstanding, we will include a clear explanation of this layout in the table notes to ensure that readers can accurately interpret the content and logical structure of the table. The specific excerpt is as follows:

*“Due to table length limitations, Table 8 divides the social integration dimension into two parts: the upper section presents the regression results of land price competition on urban-rural social integration, while the lower section displays the regression results of land fiscal dependence on urban-rural social integration.”*Please refer to lines 466 to 468 in the article for the specific details.

3.In all the tables, tell what the numbers in parentheses represent.

A：Thank you very much for your suggestion. In the notes under Table 3, we have provided a detailed explanation of the meaning of the numbers in parentheses in the table. The excerpt is as follows:

*“***, **, and * indicate significance levels at 1%, 5%, and 10%, respectively. The numbers in parentheses represent standard errors. The same applies to the following tables.”*Please refer to lines 379 to 380 in the article for the specific details.

4.The coefficients obtained in the estimated models are numerically very low. This issue is probably related to the different units of the variables. Check this item and check by changing the unit of variables.

A：Thank you very much for your thorough review. As the reviewer mentioned, this paper identified the issue of relatively low estimated coefficient values during the initial drafting stage. We also attempted various solutions, such as changing the units of variables, to address this issue. For example, the paper conducted a re-analysis of the baseline regression after modifying the units of the variables for land price competition and land fiscal dependence. The results are as follows: the estimated coefficients remained relatively low in numerical value.We believe that, although adjusting variable units can alter the numerical value of the coefficients, it does not change the actual impact of the independent variables on the dependent variables. Therefore, we consider these results to be credible and have retained the original regression results without further adjustment.

|  | (1) | (2) | (3) | (4) |
| --- | --- | --- | --- | --- |
|  | Degree | Degree | Degree | Degree |
| lpc | 0.0009*** |  | 0.0009*** | 0.0009*** |
|  | (0.0003) |  | (0.0003) | (0.0003) |
| lfd |  | -1.42e-07 ** | -1.56e-07** |  |
|  |  | (6.73e-08 ) | (6.73e-08) |  |
| （lpc2） |  |  |  | -0.0002 |
|  |  |  |  | (0.0002) |
| jck | -0.0013*** | -0.0013*** | -0.0013*** | -0.0013*** |
|  | (0.0003) | (0.0003) | (0.0003) | (0.0003) |
| rlzb | -0.0001 | -0.0001 | -0.0001 | -0.0001 |
|  | (0.0001) | (0.0001) | (0.0001) | (0.0001) |
| zfgy | -0.0030*** | -0.0031*** | -0.0031*** | -0.0030*** |
|  | (0.0008) | (0.0008) | (0.0008) | (0.0008) |
| czh | -0.0052*** | -0.0056*** | -0.0052*** | -0.0053*** |
|  | (0.0019) | (0.0019) | (0.0019) | (0.0019) |
| dlmj | -0.0077*** | -0.0075*** | -0.0076*** | -0.0077*** |
|  | (0.0008) | (0.0008) | (0.0008) | (0.0008) |
| _cons | 0.0961*** | 0.0938*** | 0.0969*** | 0.0968*** |
|  | (0.0077) | (0.0076) | (0.0077) | (0.0077) |
| N | 4215 | 4215 | 4215 | 4215 |
| r2 | 0.5624 | 0.5616 | 0.5630 | 0.5625 |
| Year fixed | Yes | Yes | Yes | Yes |
| City fixed | Yes | Yes | Yes | Yes |

5.On the other hand, somewhere in the text, it is stated that it is taken from the variables ln. While some variables are in the form of percentages or ratios and cannot be taken as ln.

A：Thank you very much for your thorough review. This paper has provided further explanation regarding the logarithmic transformation of certain variables. The specific excerpt is as follows:

*"In addition, to minimize heteroscedasticity and reduce the influence of outliers on the model, this study introduces certain variables into the equations in logarithmic form based on their magnitudes. For instance, variables such as land price competition, urbanization level, and infrastructure level are log-transformed to improve the model's fit and robustness."*Please refer to lines 330 to 334 in the article for the specific details.

6.In panel data, the unit root test and the presence or absence of cointegration in the long run between variables are necessary.

A：Thank you very much for your suggestion. In the empirical section of the paper, we have incorporated unit root and cointegration tests. The excerpt is as follows:

*Panel Unit Root and Cointegration Tests*

*To avoid spurious regression, it is first necessary to perform unit root tests on the panel data to determine its stationarity. This study employs both the IPS test and LLC test, which are commonly used for homogeneous unit root testing, to examine the stationarity of each variable in the panel data. If both tests reject the null hypothesis of a unit root, it indicates that the variable is stationary. Conversely, if the null hypothesis of a unit root is accepted, it implies that the variable is non-stationary.*

*The test results, as shown in Table 3, indicate that for all variables, the first-order differenced results reject the null hypothesis of "the existence of a unit root" at the 1% significance level. This confirms that all variables are stationary, thereby ruling out the possibility of spurious regression. However, due to the potential instability of panel data, directly applying the ordinary least squares (OLS) method may still result in spurious regression. Therefore, a panel cointegration test is performed next to analyze whether the relevant variables exhibit cointegration relationships.*

*Table 3 Results of Unit Root Test for Variables*

| Variables |  | IPS | LLC | Conclusion |
| --- | --- | --- | --- | --- |
| D(Degree) | Statistic | -25.0291*** | -16.7626*** | stable |
|  | P | 0.0000 | 0.0000 |  |
| D(lpc) | Statistic | -34.3859*** | -32.7703*** | stable |
|  | P | 0.0000 | 0.0000 |  |
| D(lfd) | Statistic | -34.1674*** | -36.0627*** | stable |
|  | P | 0.0000 | 0.0000 |  |
| D(jck) | Statistic | -21.7426*** | -5.0324*** | stable |
|  | P | 0.0000 | 0.0000 |  |
| D(rlzb) | Statistic | -2.7442*** | -63.9612*** | stable |
|  | P | 0.0030 | 0.0000 |  |
| D(zfgy) | Statistic | -31.7085*** | -28.4874*** | stable |
|  | P | 0.0000 | 0.0000 |  |
| D(czh) | Statistic | -23.0573*** | -1.5e+02*** | stable |
|  | P | 0.0000 | 0.0000 |  |
| D(dlmj) | Statistic | -25.3438*** | -31.6995*** | 平稳 |
|  | P | 0.0000 | 0.0000 |  |

*Note: D denotes first-order differencing, and *, **, *** represent significance levels of 10%, 5%, and 1%, respectively, at which the null hypothesis is rejected.*

*Based on the unit root test results, this study further conducts a cointegration test to verify the equilibrium relationship between variables. Specifically, the Pedroni test, Westerlund test, and Kao test are employed. The results indicate that the p-values of the test statistics are all less than 0.01. Therefore, the null hypothesis of no cointegration is rejected at the 1% significance level, suggesting the existence of a long-term stable cointegration relationship among the variables. This provides a solid foundation for conducting regression analysis.*

*Table 4 Cointegration test results*

| Inspection type | Inspection Statistics | Statistics | P |
| --- | --- | --- | --- |
| Pedroni | Modified Phillips-Perron t | 30.0642 | 0.0000 |
|  | Phillips-Perron t | -21.0833 | 0.0000 |
|  | Augmented Dickey-Fuller t | -16.4603 | 0.0000 |
| Westerlund | Variance ratio | 22.7037 | 0.0000 |
| Kao | Modified Dickey-Fuller t | 8.5473 | 0.0000 |
|  | Dickey-Fuller t | 8.0558 | 0.0000 |
|  | Augmented Dickey-Fuller t | 13.9056 | 0.0000 |
|  | Unadjusted modified Dickey-Fuller t | 2.6583 | 0.0039 |
|  | Unadjusted Dickey-Fuller t | 1.6721 | 0.0472 |

Please refer to lines 338 to 358 in the article for the specific details.

7.Why is the gmm model used? This model is a dynamic method and there is no evidence of this in the results table. And of course, why are there only three degrees in gmm results in table 7?

A:Thank you very much for your suggestion. The rationale for using the GMM model lies in the fact that, although GMM is a dynamic method, it is also commonly used to address endogeneity issues. In panel data analysis, urban-rural integration development may be influenced by other factors such as regional economic conditions and cultural traditions, which might have a bidirectional causal relationship with local governments' land transfer behaviors. Using conventional OLS or fixed effects models may result in biased estimation results. By leveraging instrument variables, the dynamic GMM method effectively mitigates endogeneity problems, providing more reliable estimation results. Therefore, this study adopts the GMM model, drawing on established practices in the literature, to conduct robustness tests.

Additionally, the GMM test results are presented in only three columns for the following reason: based on the theoretical framework established earlier, this study employs the system GMM estimation to empirically test three aspects: (1) the impact of land price competition on urban-rural integration, (2) the impact of land fiscal dependence on urban-rural integration, and (3) the "inverted U-shaped" relationship observed between land price competition and urban-rural integration. Consequently, the GMM results are displayed in only three columns.To further clarify the rationale for using the GMM model, we will explicitly explain its usage in the paper and elaborate on all three columns of results in the discussion section. The excerpt is as follows:

*"The GMM model is a commonly used estimation method to address endogeneity issues. Particularly in panel data analysis, urban-rural integration development may be influenced by other factors such as regional economic conditions, cultural traditions, and others, which might also have a bidirectional causal relationship with local governments' land transfer behaviors. Using conventional OLS or fixed effects models could lead to biased estimation results. The GMM estimation method, by employing instrument variables, effectively alleviates endogeneity issues, thus providing more reliable estimation results. Therefore, to obtain relatively unbiased and consistent estimation results, this study employs the system GMM estimation method to empirically examine the relationship between local governments' land transfer behaviors and urban-rural integration development (see Table 9).From column (1) in Table 9, it can be seen that the regression results for the core explanatory variable align with theoretical expectations, indicating that land price competition has a significant positive impact on urban-rural integration at the 1% significance level. Column (2) of Table 9 shows that land fiscal dependence has a significant negative impact on urban-rural integration at the 10% significance level. Column (3) of Table 9 further demonstrates that land price competition exhibits a significant "inverted U-shaped" relationship with urban-rural integration, which suggests that the regression results discussed earlier are robust."*Please refer to lines 479 to 493 in the article for the specific details.

8.List the units of the variables in a column in Table 2.

A:Thank you for your suggestion. The units of variables were not listed in Table 2 primarily due to the calculation methods of certain indicators. On the one hand, some variables are derived as ratios or proportions from quantities of the same category and therefore have no units, such as land price competition, land fiscal dependence, and labor transfer. On the other hand, certain variables originate from the computation of composite indicator systems, such as the level of urban-rural integration, which is typically obtained through entropy weighting of multidimensional variables, resulting in a dimensionless comprehensive index without a unit.Moreover, to maintain the table's simplicity and clarity, we did not list the units separately. However, in Table 2, the units are provided for variables that have them. Additionally, the definitions and calculation methods for all variables are thoroughly detailed in the data description section of the paper.

9.In line 256, the notation error term is reported incorrectly.

A:Thank you for your thorough review. We have corrected the misreported symbol$\mu_{\text{it}}$ in line 256 and replaced it with the correct symbol$\varepsilon_{\text{it}}$ Please refer to lines 250 to 251 in the article for the specific details.. Additionally, we carefully re-read the entire manuscript to ensure the avoidance of errors in symbols, writing, and other aspects. We hope these efforts contribute to improving the rigor and accuracy of the paper.

**Reply to the comments of Academic Editor**

1.The study identifies a critical research gap by focusing on the impact of local government land ‎transfer practices on urban-rural integration development, emphasizing mechanisms such as ‎land price competition and fiscal dependence. While this topic is significant and addresses a ‎policy-relevant issue in the context of China, the novelty is somewhat limited due to the ‎extensive prior work on land use and urbanization. The study's differentiation could be ‎improved by further elaborating on how its approach surpasses existing research.‎

A:Thank you very much for your suggestion. In the final paragraph of the first section, we explicitly clarified the gaps in current research and the contributions this paper seeks to make. The excerpt is as follows:

*"Existing literature predominantly focuses on the impacts of land markets, land resource allocation efficiency, and misallocation on urban-rural integration, while paying relatively little attention to the effects of local governments' land transfer behaviors on urban-rural integration. Furthermore, previous studies have not systematically conceptualized local governments' land transfer behaviors into the two dimensions of land price competition and land fiscal dependence to explore their impacts and heterogeneity concerning urban-rural integration. Additionally, the mechanisms through which local governments' land transfer behaviors influence urban-rural integration remain largely unexplored.The potential marginal contributions of this study are as follows: First, this paper is the first to systematically elucidate the impact mechanisms of local governments' land transfer behaviors on urban-rural integration, filling a gap in the existing literature. By constructing a unified theoretical framework, this study deeply examines how land transfer behaviors influence urban-rural integration through mechanisms such as labor migration, public service provision, and industrial structure upgrading. This provides a novel perspective for understanding the relationship between land transfer behaviors and urban-rural integration.Second, this study offers theoretical support and practical guidance for optimizing land transfer policies by local governments. In particular, in the context of promoting urban-rural integration, the findings of this paper help local governments better understand the multidimensional impacts of land transfer behaviors, enabling them to formulate more scientific land transfer and fiscal policies. Furthermore, the policy recommendations provided in this study can help governments rationally adjust their land transfer practices to promote labor migration, enhance public service provision, and facilitate industrial structure upgrading, thereby fostering urban-rural integration, reducing the urban-rural gap, and achieving coordinated economic and social development."*Please refer to lines 103 to 121 in the article for the specific details.

2.The paper provides a robust review of relevant literature, delineating prior studies on urban-‎rural integration and the role of land markets. However, it could better integrate more diverse ‎international perspectives to highlight potential parallels or deviations in global contexts. ‎Additionally, while the hypotheses are well-articulated, their connection to the literature could ‎be more explicitly justified.‎

A:Thank you for your suggestion. As the phenomenon of local governments' land transfer behaviors characterized by "low-priced industrial land transfers and high-priced commercial and residential land transfers" is currently unique to China, most of the existing research findings come from Chinese scholars. However, to broaden the international perspective of academic research, we have also attempted to incorporate and reference the research findings of international scholars in the discussion. For instance, in the literature review section, we have included works such as "*Kvartiuk, V., Bukin, E., and Herzfeld, T. (2024). 'For whoever has will be given more’? Land rental decisions and technical efficiency in Ukraine. Land Use Policy, 146, 107336-107336. DOI: 10.1016/J.LANDUSEPOL.2024.107336*" and "*Kalfas, D., Kalogiannidis, S., Chatzitheodoridis, F., et al. (2023). Urbanization and land use planning for achieving the sustainable development goals (SDGs): A case study of Greece. Urban Science, 7(2): 43*"*.*These references aim to enrich the international perspective of the study. The relevant content can be found in lines 96 and 97 of the paper.

In addition, following the editor's suggestion, we have enriched the theoretical analysis of the impact mechanism and included more references in support of the hypotheses to enhance their persuasiveness and clarity. The additional references are as follows:"Cheng y, Gong l, Tian wj. Industrial land transfer, factor mobility, and regional output imbalance: A perspective based on inter-regional land competition [J]. Economic Research Journal, 2024, 59(07): 170-187"、"*Li c, Jiang y. The impact of land transfer and labor migration interaction on urban-rural income disparity [J]. Business Research, 2024, (05): 81-93*"*.*"*Lan f, Xu hy, Da hl, et al. Housing prices, land transfers, and the capitalization of public services: Evidence from 284 cities in China [J]. Management Review, 2023, 35(05): 303-314*"*.*"*Zhang qh, Bian zq. The impact of the construction of industrial transformation and upgrading demonstration zones on the green utilization efficiency of urban land [J]. China Land Science, 2024, 38(10): 105-114*"*.*The relevant content can be found in lines 164. 193. 209 and 235 of the paper.

3.The use of panel data from 281 cities and bidirectional fixed effects modeling is appropriate ‎and rigorous. However, the paper could provide more clarity on the rationale for selecting ‎specific control variables and the measurement of constructs like "urban-rural integration ‎development." The robustness checks strengthen the validity, yet the discussion of potential ‎limitations in variable definitions or endogeneity concerns requires expansion.‎

A:Thank you for your suggestion. Based on the editor's feedback, this paper has more clearly explained the theoretical basis for selecting specific control variables. The specific excerpt is as follows:

*“①Degree of Openness (jck). The degree of openness of a region can impact urban-rural integration through industrial linkages and talent mobility effects. In this study, the degree of openness is measured by the ratio of total import and export volume to GDP. ②Level of Human Capital (rlzb). Urban-rural integration is significantly influenced by regional human capital factors. Human capital promotes integration by fostering creativity and enhancing the region's capacity for technological innovation. This study uses the ratio of the number of higher education students enrolled to the total year-end population to represent the level of human capital. ③Degree of Government Intervention (zfgy). Under the context of the urban-rural dual structure, local governments' fiscal expenditures often exhibit an urban bias, which affects urban-rural integration. This study measures the degree of government intervention using the ratio of local government fiscal expenditures to GDP. ④Urbanization Level (czh). Urbanization influences urban-rural integration through effects such as population migration and factor mobility. The urbanization level is measured in this study by the urbanization rate of each region. ⑤Infrastructure Level (dlmj). Improved infrastructure facilitates population inflows and industrial development in a region, thereby impacting urban-rural integration. This study measures the infrastructure level using the per capita road area of the region.”*Please refer to lines 309 to 322 in the article for the specific details.

In response to the editor's suggestions, this paper has enriched the discussion on robustness tests based on the original analysis. A summary of the additions is as follows:

*“To ensure the credibility and reliability of the findings, given that baseline regression results may be influenced by factors such as sample selection bias, variable selection errors, and endogeneity issues, this study employed multiple robustness testing methods. First, to mitigate potential endogeneity issues, the system GMM estimation was applied, using instrumental variables to replace endogenous variables and reduce potential bias. Second, to control for biases that may arise from sample heterogeneity, this study excluded samples from municipalities and re-ran the regression analysis. Third, the study replaced the measurement method of the dependent variable to verify the consistency of the regression results under different variable definitions, thereby improving the rigor of the analysis. Lastly, to further test the robustness of the findings, this study introduced lagged explanatory variables to examine the impact of dynamic effects on the regression results. ”*Please refer to lines 470 to 478 in the article for the specific details.

*“To verify the accuracy of the previous regression results, this study further employed principal component analysis (PCA) to re-measure the level of urban-rural integration and conducted regression analysis based on this revised measurement, with the results shown in Table 10, regression column (2). By extracting the core characteristic variables of urban-rural integration through PCA, this method reduces the impact of potential multicollinearity and measurement errors caused by variable selection, thereby making the measurement results more objective and comprehensive. The regression results indicate that the estimated coefficient of land price competition remains significantly positive, while the estimated coefficient of land fiscal dependence remains significantly negative. These results are consistent with the findings in the earlier sections, further validating the robustness of the impact of local governments' land transfer behaviors on urban-rural integration.”*Please refer to lines 502 to 511 in the article for the specific details.

*“Considering that land development and utilization may have a certain lag effect, and that the impact of local governments' land transfer behaviors on urban-rural integration may exhibit a time delay, this study introduced one-period lagged explanatory variables to further validate the robustness of the empirical results. The regression results are shown in Table 10, regression column (3). Here, L.lpc and L.lfd respectively represent the one-period lagged values of land price competition and land fiscal dependence. The results indicate that the estimated coefficient of land price competition remains significantly positive, suggesting that the role of land price competition in promoting urban-rural integration is sustained over time. Meanwhile, the estimated coefficient of land fiscal dependence remains significantly negative, further demonstrating the robustness of its suppressive effect on urban-rural integration.”*Please refer to lines 512 to 520 in the article for the specific details.

4.The statistical results are presented systematically, with clear interpretation of coefficients and ‎significant effects. However, the justification for the inverted U-shaped relationship between ‎land price competition and integration development could be more thoroughly grounded in ‎empirical patterns or theoretical reasoning.‎

A:Thank you very much for your thorough review. This paper provides a more detailed theoretical reasoning on the inverted U-shaped relationship between land price competition and urban-rural integration, as outlined below:

*“On the one hand, local governments adopting low or even zero-price policies for industrial land transfers can reduce the input costs for enterprises acquiring land, attracting investments from industrial and manufacturing enterprises while providing them with space for development. However, excessively low land transfer prices may lead to a "race to the bottom" among regions. As the industrial land transfer price increases, it not only raises the entry threshold for enterprises—preventing the clustering of low-efficiency or mid- to low-end enterprises—but also incentivizes enterprises to upgrade machinery, adopt advanced production technologies, and enhance operational efficiency. These dynamics are conducive to promoting the flow of resources and factors between urban and rural areas, thereby advancing urban-rural integration.However, further increases in industrial land transfer prices may bring counterproductive effects. On the one hand, higher land prices raise the entry threshold for enterprises, causing some small and medium-sized enterprises (SMEs) to lose the ability to enter the market altogether. This reduction in market diversity and competitiveness weakens the overall dynamism of the economy. On the other hand, increased land prices impose higher costs on enterprises, discouraging investment. This weakens regional economic vitality, limits the inflow of factors such as capital, technology, and human resources, and restricts the mobility of regional resources. Ultimately, these constraints hinder the optimal allocation of factors between urban and rural areas and obstruct the process of urban-rural integration.This reasoning provides a theoretical explanation for the observed inverted U-shaped relationship between land price competition and urban-rural integration, illustrating the dual impact of land price increases on resource allocation and economic development.”* Please refer to lines 148 to 165 in the article for the specific details.

5.The study makes a meaningful theoretical contribution by incorporating labor transfer, public ‎services, and industrial structure upgrading into a unified framework. Nonetheless, it would ‎benefit from a deeper discussion of how these mechanisms extend or challenge existing ‎theories on urban-rural dynamics.‎

A：Thank you very much for your suggestion. In the introduction section, we have elaborated on the theoretical and practical significance of this study in the current research field, rather than providing a general overview. The excerpt is as follows:

*“The potential marginal contributions of this study are as follows: First, this paper systematically elucidates, for the first time, the impact mechanisms of local governments' land transfer behaviors on urban-rural integration, filling a gap in the existing literature. By constructing a unified theoretical framework, this study delves into how land transfer behaviors influence urban-rural integration through pathways such as labor migration, public service provision, and industrial structure upgrading. This provides a novel perspective for theories related to land transfer behaviors and urban-rural integration. Second, this research offers theoretical support and practical guidance for optimizing land transfer policies by local governments. Particularly in the context of promoting urban-rural integration, the findings of this paper help local governments better understand the multidimensional impacts of land transfer behaviors, enabling them to formulate more scientific land transfer and fiscal policies. Furthermore, the policy recommendations proposed in this study assist governments in rationally adjusting land transfer practices to facilitate labor migration, enhance public service provision, and promote industrial structure upgrading. In doing so, the study aims to foster urban-rural integration, reduce urban-rural disparities, and achieve coordinated economic and social development.”* Please refer to lines 109 to 121 in the article for the specific details.

6.While the paper provides actionable insights, such as the need for regional customization of ‎land policies, its recommendations could be more nuanced. For instance, proposing strategies ‎for regions with high land finance dependence beyond reducing reliance could add depth to the ‎practical implications.‎

A:Thank you very much for your suggestions. This paper has made more detailed and nuanced modifications to the recommendations section, as outlined below:

*“Therefore, local governments need to find a reasonable balance in the pricing of industrial land transfers. To reduce land costs for enterprises and attract high-quality external enterprises, local governments can explore diversified land supply mechanisms, such as land leasing, "rent-first, sell-later" schemes, and long-term leases. These approaches not only alleviate the initial investment pressure on enterprises but also allow for more flexible adjustments to the efficiency of land resource allocation. Based on this, governments can guide high-quality enterprises to form industrial chain effects through agglomeration, promote the scaled development of urban economies, and leverage the city's role in driving rural areas. This facilitates the optimized flow of economic factors between urban and rural areas, achieving the long-term sustainability of urban-rural integration.At the same time, local governments should gradually reduce their heavy reliance on land-based fiscal revenues and promote the diversification of fiscal revenue sources. For example, governments can develop emerging industries, expand regional financing channels, and advance public-private partnership (PPP) models to increase fiscal revenue sources. Additionally, strict oversight of land transfer revenue utilization is necessary, with funds prioritized for urban-rural infrastructure development, the equalization of public services, and rural revitalization initiatives. This helps avoid the negative effects of land-based finance, such as high housing prices and homogeneous industrial structures, which hinder urban-rural integration. In this way, a solid foundation for coordinated urban-rural economic development can be established.”* Please refer to lines 597 to 613 in the article for the specific details.

*“Regarding land fiscal dependence, western regions and cities with lower administrative levels need to reduce biased expenditures driven by land-based fiscal revenues and reallocate these resources toward infrastructure, healthcare, education, and other public services. Increasing the supply of affordable housing is also essential to alleviate pressure on housing prices. For regions highly dependent on land-based fiscal revenues, strategies such as developing diversified fiscal revenue sources, promoting characteristic and green industries, optimizing land revenue utilization models, and establishing interregional fiscal balance mechanisms can gradually reduce over-reliance on land transfer revenues and foster urban-rural integration.”* Please refer to lines 619 to 626 in the article for the specific details.

*“On one hand, local governments should increase investment in regional infrastructure development, improving transportation networks, information and communication systems, and other physical infrastructure. At the same time, optimizing public services such as social security and housing benefits can enhance cities' inclusiveness and attractiveness to rural labor forces, facilitating the free flow of labor factors between urban and rural areas and promoting optimized resource allocation. On the other hand, local governments should focus on improving human capital levels by strengthening vocational education and skills training. This enhances the overall quality and adaptability of the labor force, while policy guidance can direct labor flows toward advantageous and emerging industries, achieving regional industrial structure optimization and upgrading. These measures strengthen economic endogenous growth and further promote urban-rural integration.”* Please refer to lines 631 to 639 in the article for the specific details.

Once again, thank you very much for your comments and suggestions! Those comments are all valuable and very helpful for revising and improving our paper.

Best Wishes!
