# Supplementary material for: Research on the impact and mechanism of local governments’ land conveyance behavior on urban-rural integrated development—Empirical evidence from 281 prefecture-level cities in China
Source: PLoS One. 2025 Jul 7;20(7):e0327433. doi: 10.1371/journal.pone.0327433 (PMC12233302; doi:10.1371/journal.pone.0327433)
Supplement: Attachment — Submitted filename: Comment for Plos Journal.docx [file pone.0327433.s003.docx]

Comments

Here are the strengths of the manuscript: the issue is interesting and eligible for academic discussion, with major variables identified in the numeric analysis. However, there are several areas that need improvement:

1. The manuscript lacks clarity in its preparation.
2. It does not provide sufficient depth and breadth in the theoretical and conceptual framework.
3. The literature review section needs to be reworked to address topical issues and include relevant empirical studies.
4. The research methodology (approach, design, data gathering tools, sampling, analysis, etc.) is not adequately described.
5. The research discussion is not supported by empirical evidence or other literature; it lacks triangulation techniques.
6. The study lacks qualitative data sources and their analysis, focusing solely on numerical data. Therefore, the manuscript is not suitable for policy research.
7. The manuscript contains redundancy of ideas throughout the paper.
8. The title of the manuscript requires modification.
