# Supplementary material for: Research on the impact and mechanism of local governments’ land conveyance behavior on urban-rural integrated development—Empirical evidence from 281 prefecture-level cities in China
Source: PLoS One. 2025 Jul 7;20(7):e0327433. doi: 10.1371/journal.pone.0327433 (PMC12233302; doi:10.1371/journal.pone.0327433)
Supplement: Attachment — Submitted filename: Response_to_Reviewers_auresp_2.docx [file pone.0327433.s004.docx]

**Manuscript ID:**PONE-D-24-33248

**Title:**  Research on the Impact and Mechanism of Local Government Land Transfer Behavior on Urban Rural Integration Development——Perspectives based on land price competition and land finance dependence

**Authors name:**Long Zeng , Bin Peng' * and Jin Xie

**Response to Reviewers**

Dear Editor and Reviewers,

Thanks very much for taking your time to review this manuscript. we really appreciate all your comments and suggestions! Below, we provide a detailed response to each comment raised by the reviewers and outline the revisions made accordingly. In addition, the modifications we have made have been highlighted in red font in the re-submitted files.

**Reply to the comments of Reviewer 4**

1.The literature review is not sufficiently processed, even though it is a case study, it would be appropriate to supplement the review with the results of research on the given issue from other countries.

A:Thank you for your valuable suggestions. The literature review section of this paper has been revised to incorporate additional research findings from international scholars. Such as “*SANSO M, 2011. Urban agglomeration and the aggregate economic growth. European Regional Science Association.*”、“*Church P S ,Floress M K ,Ulrich-Schad D J , et al. How water quality improvementefforts influence urban–agricultural relationships [J]. Agriculture and Human Values,2020: 1-18.*”、“*Katrin M ,Sebastian R ,Jana Z , et al. Classifying New Hybrid Cooperation Models for Short Food-Supply Chains—Providing a Concept for Assessing Sustainability Transformation in the Urban-Rural Nexus [J]. Land, 2022, 11 (4): 582-582.*”、“*Leonard M S, Kerre P M, Boniface E E. Rural-urban interlink and sustainability of urban centresin Kenya: a case of Malaba Town[J]. Journal of US-China Public Administration, 2011,8(8):871-883*.”、“*Liu y s, Cong s, Li y r. Differentiation Regularity of Urban-Rural Equalized Development at PrefectureLevel City in China［J］. Journal of Geographical Sciences，2015，25（09）：1075-1088*.”、“*Zhao y j. Land finance: History, logic, and policy choices. Urban Development Studies, 2014(1), 1–13.*” and “*Li s, Yang y x. Equalization of basic public services for common prosperity: Action logic and pathway selection. China Industrial Economics,2022 (2), 27–41.*”

The specific summary of the literature review is as follows:

*“Under the urban-rural dual structure, cities typically offer more employment opportunities and higher wages, which incentivize the migration of labor and other production factors from rural to urban areas. However, with the evolving development environment between urban and rural regions, a reverse trend has emerged, where production factors, including labor, are increasingly flowing back from cities to rural areas. Urban-rural integrated development is widely regarded as a process that facilitates the rational flow and optimal allocation of production factors between urban and rural areas, thereby promoting economic integration and interactive growth. Existing literature has explored the conceptual framework and practical challenges of urban-rural integration from a theoretical perspective [5], while also identifying its driving mechanisms and pathways [6]. Empirical studies have further measured the level of urban-rural integration and examined its influencing factors from various dimensions [7], including economic growth [8], economic agglomeration [9], misallocation of urban-rural production factors [10], factor mobility [11], water quality improvement measures [12], new industrial cooperation models [13], and infrastructure development [14]. These studies often adopt analytical units at the provincial or prefecture-city level [15].”* For specific details, please refer to lines 77 to 89 of the manuscript.

2.what basis are high prices defined？

A: We sincerely appreciate your thorough review. Regarding the definitions of "low-price" industrial land and "high-price" commercial/residential land, this study is grounded in China’s distinctive land conveyance system and practices. After extensive literature review, we adopt the established consensus in Chinese land economics research: local governments deliberately lease industrial land at low prices to attract investment while auctioning commercial/residential land at high prices to generate fiscal revenue for regional development. This dual pricing strategy accurately reflects their "land-driven development" behavior. Key excerpts are summarized below:

“*As a critical foundation for regional economic and social development, land serves as a major fiscal revenue source for local governments under China's land concession system and the fiscal decentralization framework characterized by "centralized fiscal authority but decentralized administrative responsibilities. "With local governments holding absolute control over land concessions, land leasing and financing have become vital in supporting regional economic development and industrial policy formulation [35]. This model follows a dual institutional logic: On the one hand, Driven by political promotion incentives, local governments attract investment by leasing industrial land at low prices, creating employment opportunities and boosting economic growth [36]. For example, Jiangsu Province’s 2021 Opinions on Further Promoting Industrial Land Efficiency emphasizes that industrial land is essential for sustaining real economic development—whether for upgrading traditional manufacturing or establishing new industrial bases. On the other hand, Under fiscal pressure from revenue-expenditure imbalances, local governments generate substantial income by leasing commercial/residential land at higher prices. This helps alleviate fiscal strain from decentralization while funding infrastructure projects [3]. For instance, Chaoyang City, Liaoning Province’s 2021 budget report showed municipal government-managed fund revenues reaching ¥10.294 billion (103% of the target), a 131.7% increase—primarily due to rising real estate development activity and higher land concession revenues.*”For specific details, please refer to lines 127 to 141 of the manuscript.

3.Why are relatively old data used

A：We sincerely appreciate your thorough review. The study’s sample period ends in 2017 because the China Land and Resources Statistical Yearbook, which publishes official land transaction price data, ceased publication after that year. Consequently, our empirical analysis is necessarily limited to pre-2018 data. The specific data sources and methodological explanations are excerpted below:

*“This study employs panel data of Chinese prefecture-level and above cities from 2003 to 2017. The initial sample included all prefecture-level cities nationwide. However, to ensure research consistency and comparability, we excluded certain cities (e.g., Lhasa, Zhongwei, Chaohu, and Bijie) due to data deficiencies or administrative division adjustments, ultimately retaining a balanced panel of 281 cities. Notably, the study period concludes in 2017 as the primary data source - China Land and Resources Yearbook - ceased publication thereafter, making subsequent land transaction price data unavailable. ”*For specific details, please refer to lines 330 to 335 of the manuscript.

4.what basis was the sample of cities selected?

A：We sincerely appreciate your reviewers’ insightful comments. This study utilizes panel data covering all 293 prefecture-level cities in China from 2003 to 2017. The dataset adopts city-wide administrative boundaries (rather than urban districts only) to ensure rural areas are incorporated—a necessary approach given our focus on urban-rural integrated development. However, due to missing data or administrative boundary adjustments (e.g., Lhasa, Zhongwei, Chaohu, and Bijie), the final sample comprises 281 prefecture-level cities. Detailed data sources and screening criteria are provided below:

*“This study employs panel data of Chinese prefecture-level and above cities from 2003 to 2017. The initial sample included all prefecture-level cities nationwide. However, to ensure research consistency and comparability, we excluded certain cities (e.g., Lhasa, Zhongwei, Chaohu, and Bijie) due to data deficiencies or administrative division adjustments, ultimately retaining a balanced panel of 281 cities. Notably, the study period concludes in 2017 as the primary data source - China Land and Resources Yearbook - ceased publication thereafter, making subsequent land transaction price data unavailable. ”*For specific details, please refer to lines 330 to 335 of the manuscript.

5. In the conclusion, the research limits are missing.

A:We sincerely appreciate your thoughtful review of our manuscript. In response, we have significantly restructured this paper by adding a dedicated Discussion section. This new section not only strengthens critical engagement with findings but also explicitly highlights key limitations of this study. Relevant details are excerpted below:

*“Furthermore, while this study systematically evaluates the impact of local governments’ land conveyance behaviors on urban-rural integration, it may still have the following limitations: (1) In constructing the urban-rural integration development index system, due to the challenges of measurement and processing complexity, this paper, drawing on existing literature, only selected partial data from sources such as statistical yearbooks, prefecture-level city statistical yearbooks, the China Urban and Rural Construction Database, and the China Regional Economic Database. This approach may introduce slight deviations from the actual level of urban-rural integration. Additionally, the selected indicators are limited to quantitative metrics from statistical yearbooks and similar sources, excluding qualitative factors such as government institutions, governance structures, and officials’ service awareness. (2) Although variables such as the degree of openness to the outside world and human capital levels were controlled for, other potential influencing factors—such as local policy preferences, historical development trajectories, and natural geographical conditions—may have been overlooked in their impact on urban-rural integration. (3) This study only examined the moderating effects in the relationship between local governments’ land conveyance behaviors and urban-rural integration, without analyzing whether these behaviors indirectly influence urban-rural integration through mediating variables.”*For specific details, please refer to lines 602 to 615 of the manuscript.

**Reply to the comments of Reviewer 5**

1.The manuscript lacks clarity in its preparation.

A：Thank you very much for your suggestions. In response to this issue, under the guidance of professional instructors, this paper has standardized the writing expression to enhance its logical flow, improve language coherence, and ensure compliance with academic writing norms. Additionally, the structure of the paper has been refined, and a discussion section has been added to better meet the requirements for publication in academic journals. Specific excerpts are as follows:

*Discussion*

*“The integration of urban and rural development is a crucial measure for advancing rural revitalization, with local government land transfer practices serving as a key link in promoting this integration. Based on panel data from 281 prefecture-level and above cities in China, this study constructs a two-way fixed effects model to categorize local government land transfer behaviors into two dimensions: low-price industrial land transfers and high-price commercial and residential land transfers, integrating them into a unified theoretical framework. The study systematically evaluates the impact of local government land transfer behaviors on urban-rural integration and conducts an empirical analysis of their effects and mechanisms. The research selects land price competition and fiscal dependence on land as core explanatory variables, employs the entropy method to calculate a comprehensive index of urban-rural integration as the dependent variable, and controls for variables such as the degree of openness, human capital level, and government intervention. In terms of mechanism testing, a moderating effects model is used to analyze the moderating effects of labor migration, public service provision, and industrial structure upgrading. The study also examines heterogeneous impacts across regions, administrative levels, and dimensions of urban-rural integration through subgroup regressions. Robustness checks are conducted using methods such as system GMM estimation, alternative measures of the dependent variable, and lagged explanatory variables. The key findings are as follows: (1) Among local government land transfer practices, land price competition exerts a significant positive effect on urban-rural integration while simultaneously exhibiting an inverted U-shaped relationship with it. (2) Fiscal dependence on land, however, inhibits urban-rural integration. (3) The impact of local government land transfer behavior on urban-rural integration exhibits heterogeneity across regions, administrative levels, and different dimensions of integration. (4) Mechanism analysis reveals that labor migration, public service provision, and industrial structure upgrading serve as critical channels through which local governments' land transfer behaviors influence urban-rural integration. Specifically, Labor migration and industrial structure upgrading strengthen the positive effect of land price competition; Public service provision and industrial structure upgrading mitigate the negative impact of fiscal dependence on land.*

*Compared to prior studies, this paper draws extensively on key insights from the literature regarding the relationship between land markets—such as land resource misallocation [10] and factor mobility [11]—and urban-rural integration. It also references empirical methods for measuring urban-rural integration levels, land marketization, and econometric model construction [51], while extending the theoretical framework on how land market reforms shape urban-rural dynamics [24]. Our findings confirm that land price competition positively contributes to urban-rural integration, whereas fiscal dependence on land exerts a negative effect. However, some scholars argue that land finance serves as a crucial engine for regional economic development [59]. Thus, dismissing its role entirely would be unbalanced; instead, efforts should focus on diversifying local government revenue sources to gradually reduce reliance on land finance and achieve sustainable urban-rural integration. Notably, this study exhibits significant distinctions from existing research in the following aspects: First, regarding research perspective, prior literature predominantly focuses on the impact of land markets per se or land resource allocation efficiency on urban-rural integration [29]. In contrast, this paper centers on local governments' land conveyance behaviors, internalizing them into two dimensions—land price competition and fiscal dependency on land revenue—thereby offering deeper insights into the mechanisms through which local governments' land transactions influence urban-rural integration. Second, existing studies posit that high-quality public service provision fosters a conducive environment for enterprises and investors, facilitating the entry and agglomeration of external firms and thereby promoting regional industrial upgrading and economic development [60]. However, our analysis reveals that public service provision does not exhibit a significant positive moderating effect in the relationship between local governments’ land conveyance behaviors and urban-rural integration. A plausible explanation lies in the fact that while local governments’ low-price industrial land transfers may attract more enterprises and capital, stimulating economic growth and employment opportunities, they simultaneously necessitate increased public service investments (e.g., infrastructure) to sustain such development. Yet, the public services provided may fail to adequately support the ancillary needs of enterprises attracted by underpriced industrial land, thereby diminishing the anticipated reinforcing effect of public services on urban-rural integration under conditions of land price competition. Conversely, the interaction term between public service provision and fiscal dependency on land revenue shows a significantly positive coefficient. This suggests that enhancing public service levels—such as infrastructure and social security investments—can facilitate cross-regional flows of labor and other resources. Such improvements effectively mitigate adverse effects stemming from fiscal reliance on land revenue (e.g., soaring housing prices, industrial rigidity, and homogenization), thereby weakening its negative impact on urban-rural integration.*

*Furthermore, while this study systematically evaluates the impact of local governments’ land conveyance behaviors on urban-rural integration, it may still have the following limitations: (1) In constructing the urban-rural integration development index system, due to the challenges of measurement and processing complexity, this paper, drawing on existing literature, only selected partial data from sources such as statistical yearbooks, prefecture-level city statistical yearbooks, the China Urban and Rural Construction Database, and the China Regional Economic Database. This approach may introduce slight deviations from the actual level of urban-rural integration. Additionally, the selected indicators are limited to quantitative metrics from statistical yearbooks and similar sources, excluding qualitative factors such as government institutions, governance structures, and officials’ service awareness. (2) Although variables such as the degree of openness to the outside world and human capital levels were controlled for, other potential influencing factors—such as local policy preferences, historical development trajectories, and natural geographical conditions—may have been overlooked in their impact on urban-rural integration. (3) This study only examined the moderating effects in the relationship between local governments’ land conveyance behaviors and urban-rural integration, without analyzing whether these behaviors indirectly influence urban-rural integration through mediating variables.*”For specific details, please refer to lines 548 to 615 of the manuscript.

2.It does not provide sufficient depth and breadth in the theoretical and conceptual framework.

A：Thank you for your valuable suggestions. This paper has revised the theoretical analysis section, further supplementing the institutional logic behind the characteristic facts of local governments' land transfer behaviors. It enriches the conceptual framework of local governments' land transfer practices to enhance the depth and breadth of the theoretical system. Specific excerpts are as follows:

*“As a critical foundation for regional economic and social development, land serves as a major fiscal revenue source for local governments under China's land concession system and the fiscal decentralization framework characterized by "centralized fiscal authority but decentralized administrative responsibilities. "With local governments holding absolute control over land concessions, land leasing and financing have become vital in supporting regional economic development and industrial policy formulation [35]. This model follows a dual institutional logic: On the one hand, Driven by political promotion incentives, local governments attract investment by leasing industrial land at low prices, creating employment opportunities and boosting economic growth [36]. For example, Jiangsu Province’s 2021 Opinions on Further Promoting Industrial Land Efficiency emphasizes that industrial land is essential for sustaining real economic development—whether for upgrading traditional manufacturing or establishing new industrial bases. On the other hand, Under fiscal pressure from revenue-expenditure imbalances, local governments generate substantial income by leasing commercial/residential land at higher prices. This helps alleviate fiscal strain from decentralization while funding infrastructure projects [3]. For instance, Chaoyang City, Liaoning Province’s 2021 budget report showed municipal government-managed fund revenues reaching ¥10.294 billion (103% of the target), a 131.7% increase—primarily due to rising real estate development activity and higher land concession revenues.”*For specific details, please refer to lines 127 to 141 of the manuscript.

3.The literature review section needs to be reworked to address topical issues and include relevant empirical studies.

A:Thank you for your valuable suggestions. The literature review section of this paper has been revised to incorporate additional research findings from international scholars. Such as “*SANSO M, 2011. Urban agglomeration and the aggregate economic growth. European Regional Science Association.*”、“*Church P S ,Floress M K ,Ulrich-Schad D J , et al. How water quality improvementefforts influence urban–agricultural relationships [J]. Agriculture and Human Values,2020: 1-18.*”、“*Katrin M ,Sebastian R ,Jana Z , et al. Classifying New Hybrid Cooperation Models for Short Food-Supply Chains—Providing a Concept for Assessing Sustainability Transformation in the Urban-Rural Nexus [J]. Land, 2022, 11 (4): 582-582.*”、“*Leonard M S, Kerre P M, Boniface E E. Rural-urban interlink and sustainability of urban centresin Kenya: a case of Malaba Town[J]. Journal of US-China Public Administration, 2011,8(8):871-883*.”、“*Liu y s, Cong s, Li y r. Differentiation Regularity of Urban-Rural Equalized Development at PrefectureLevel City in China［J］. Journal of Geographical Sciences，2015，25（09）：1075-1088*.”、“*Zhao y j. Land finance: History, logic, and policy choices. Urban Development Studies, 2014(1), 1–13.*” and “*Li s, Yang y x. Equalization of basic public services for common prosperity: Action logic and pathway selection. China Industrial Economics,2022 (2), 27–41.*”

The specific summary of the literature review is as follows:

*Under the urban-rural dual structure, cities typically offer more employment opportunities and higher wages, which incentivize the migration of labor and other production factors from rural to urban areas. However, with the evolving development environment between urban and rural regions, a reverse trend has emerged, where production factors, including labor, are increasingly flowing back from cities to rural areas. Urban-rural integrated development is widely regarded as a process that facilitates the rational flow and optimal allocation of production factors between urban and rural areas, thereby promoting economic integration and interactive growth. Existing literature has explored the conceptual framework and practical challenges of urban-rural integration from a theoretical perspective [5], while also identifying its driving mechanisms and pathways [6]. Empirical studies have further measured the level of urban-rural integration and examined its influencing factors from various dimensions [7], including economic growth [8], economic agglomeration [9], misallocation of urban-rural production factors [10], factor mobility [11], water quality improvement measures [12], new industrial cooperation models [13], and infrastructure development [14]. These studies often adopt analytical units at the provincial or prefecture-city level [15].* For specific details, please refer to lines 77 to 89 of the manuscript.

4.The research methodology (approach, design, data gathering tools, sampling, analysis, etc.) is not adequately described.

A：Thank you for your valuable suggestions. This paper provides a more detailed explanation and introduction of the two-way fixed effects model and the moderation effect model in the methodology section. Additionally, the discussion section elaborates on the research approach of this study. Specific excerpts are as follows:

(1)Regarding the introduction of research methodologies:

*“Based on theoretical analysis and research hypotheses, this study employs a two-way fixed effects model to systematically examine the impact of local governments' land conveyance behavior on urban-rural integrated development. The model simultaneously controls for individual fixed effects and time fixed effects, effectively addressing estimation biases caused by time-invariant regional heterogeneity and region-invariant temporal factors. The specific model is specified as follows:”*For specific details, please refer to lines 246 to 250 of the manuscript.

*“Furthermore, given the significant interactive relationships between local government land conveyance behavior and factors such as labor mobility, public service provision, and industrial structure upgrading—which may influence its impact on urban-rural integration—this study extends the baseline model by incorporating interaction terms. This approach allows for an examination of whether the effect of land conveyance is moderated by these channels. The extended moderated effects model is specified as follows:”*For specific details, please refer to lines 257 to 261 of the manuscript.

(2)Regarding the elaboration of the research approach:

*“The integration of urban and rural development is a crucial measure for advancing rural revitalization, with local government land transfer practices serving as a key link in promoting this integration. Based on panel data from 281 prefecture-level and above cities in China, this study constructs a two-way fixed effects model to categorize local government land transfer behaviors into two dimensions: low-price industrial land transfers and high-price commercial and residential land transfers, integrating them into a unified theoretical framework. The study systematically evaluates the impact of local government land transfer behaviors on urban-rural integration and conducts an empirical analysis of their effects and mechanisms. The research selects land price competition and fiscal dependence on land as core explanatory variables, employs the entropy method to calculate a comprehensive index of urban-rural integration as the dependent variable, and controls for variables such as the degree of openness, human capital level, and government intervention. In terms of mechanism testing, a moderating effects model is used to analyze the moderating effects of labor migration, public service provision, and industrial structure upgrading. The study also examines heterogeneous impacts across regions, administrative levels, and dimensions of urban-rural integration through subgroup regressions. Robustness checks are conducted using methods such as system GMM estimation, alternative measures of the dependent variable, and lagged explanatory variables. ”*For specific details, please refer to lines 549 to 563 of the manuscript.

5.The research discussion is not supported by empirical evidence or other literature; it lacks triangulation techniques.

A：Thank you for your valuable suggestions. This paper has refined the overall structure by enhancing the discussion section with additional empirical data support in the results analysis, while also incorporating relevant literature and policy documents to strengthen the discussion. Specific excerpts are as follows:

(1) Regarding empirical data support in the analysis:

*“The empirical results reveal significant regional heterogeneity: the coefficient of land price competition in western China is significantly positive at the 1% level, whereas the estimated coefficients for eastern and central regions are statistically insignificant. These findings indicate that land price competition in western China has a significant positive effect on urban-rural integrated development, while no significant impact is observed in eastern and central regions”* For specific details, please refer to lines 393 to 397 of the manuscript.

*“The regression results in Column (1) of Table 9 confirm that land price competition has a significantly positive effect on urban-rural integrated development at the 1% significance level, consistent with theoretical expectations. Column (2) further reveals a significant inverted-U-shaped relationship, indicating that while moderate land price competition promotes urban-rural integration, excessive competition may eventually weaken its positive effects. Additionally, Column (3) shows that fiscal reliance on land revenue has a significantly negative impact at the 10% level, reinforcing the robustness of our earlier findings. These results collectively validate our core conclusions regarding the differential effects of land market policies across regions.”* For specific details, please refer to lines 496 to 503 of the manuscript.

*“Considering that municipalities directly under central government administration may exhibit significant differences in urban-rural integration development due to their economic characteristics, this study excludes sample data from Beijing, Tianjin, Shanghai, and Chongqing for regression estimation. The results, as shown in Regression (1) of Table 10. The empirical results demonstrate that land price competition has a significantly positive effect on urban-rural integrated development at the 1% significance level, while fiscal reliance on land revenue exerts a significantly negative impact at the 10% level. These findings align with theoretical expectations, confirming the robustness of the baseline regression estimates.”* For specific details, please refer to lines 505 to 511 of the manuscript.

*“To verify the accuracy of the previous regression results, this study further employed principal component analysis (PCA) to re-measure the level of urban-rural integration and conducted regression analysis based on this revised measurement, with the results shown in Table 10, regression column (2). By extracting the core characteristic variables of urban-rural integration through PCA, this method reduces the impact of potential multicollinearity and measurement errors caused by variable selection, thereby making the measurement results more objective and comprehensive. The regression results indicate that the coefficient of land price competition is significantly positive at the 1% level, while the coefficient of fiscal reliance on land revenue is significantly negative at the 1% level. These findings are consistent with previous conclusions, further validating that local governments' land transfer behaviors exert robust and divergent effects on urban-rural integrated development.”* For specific details, please refer to lines 512 to 521 of the manuscript.

*“Considering that land development and utilization may have a certain lag effect, and that the impact of local governments' land transfer behaviors on urban-rural integration may exhibit a time delay, this study introduced one-period lagged explanatory variables to further validate the robustness of the empirical results. The regression results are shown in Table 10, regression column (3). Here, L.lpc and L.lfd respectively represent the one-period lagged values of land price competition and land fiscal dependence. The regression results reveal two key insights: The coefficient of land price competition is significantly negative at the 1% level, suggesting its role in promoting urban-rural integrated development exhibits a persistent influence over time rather than being limited to short-term effects; The coefficient of fiscal reliance on land revenue remains significantly negative at the 1% level, further confirming earlier findings that excessive dependence on land finance inhibits urban-rural integration—a result demonstrating robust consistency across analyses.*” For specific details, please refer to lines 522 to 532 of the manuscript.

(2) Regarding literature-based support in the discussion section:

*“Furthermore, as shown in Regression (4), the significantly negative coefficient of the quadratic term for land price competition confirms an inverted U-shaped relationship between land price competition and urban-rural integrated development. This finding theoretically aligns with the threshold effect of industrial land transfers documented by Yang et al.[36], while providing empirical support for our earlier Hypothesis H1.”*For specific details, please refer to lines 377 to 381 of the manuscript.

*“The regression results on fiscal reliance on land revenue indicate that the estimated coefficient for western China is significantly negative at the 1% level, while that of eastern China is statistically insignificant and central China shows a significantly positive coefficient. This suggests that fiscal dependence on land sales in western regions exerts a significantly negative effect on urban-rural integrated development, whereas it has a positive impact in central regions and no significant effect in eastern regions. A plausible explanation lies in regional economic disparities: In western China, underdeveloped economies with lower household incomes and consumption levels discourage diversified corporate investments[57]. This exacerbates structural rigidity and industrial homogenization caused by local governments' reliance on high-priced commercial/residential land sales, hindering urban-rural integration. In central China, rapid economic growth aligns with the State Council’s 2021 Guidelines on Promoting High-Quality Development in the New Era, which emphasize building an advanced manufacturing-led modern industrial system. Local governments thus incentivize infrastructure investments using land-sale revenues to facilitate industrial relocation from eastern regions. ”* For specific details, please refer to lines 406 to 417 of the manuscript.

6.The study lacks qualitative data sources and their analysis, focusing solely on numerical data. Therefore, the manuscript is not suitable for policy research.

A：Thank you for your valuable suggestions. To enhance the scientific rigor and persuasiveness of the theoretical analysis, this study has incorporated qualitative documents such as policy papers and statistical bulletins into the theoretical analysis section. Specific excerpts are as follows:

*“This model follows a dual institutional logic: On the one hand, Driven by political promotion incentives, local governments attract investment by leasing industrial land at low prices, creating employment opportunities and boosting economic growth [36]. For example, Jiangsu Province’s 2021 Opinions on Further Promoting Industrial Land Efficiency emphasizes that industrial land is essential for sustaining real economic development—whether for upgrading traditional manufacturing or establishing new industrial bases. On the other hand, Under fiscal pressure from revenue-expenditure imbalances, local governments generate substantial income by leasing commercial/residential land at higher prices. This helps alleviate fiscal strain from decentralization while funding infrastructure projects [3]. For instance, Chaoyang City, Liaoning Province’s 2021 budget report showed municipal government-managed fund revenues reaching ¥10.294 billion (103% of the target), a 131.7% increase—primarily due to rising real estate development activity and higher land concession revenues.”* For specific details, please refer to lines 131 to 141 of the manuscript.

*“ In central China, rapid economic growth aligns with the State Council’s 2021 Guidelines on Promoting High-Quality Development in the New Era, which emphasize building an advanced manufacturing-led modern industrial system. Local governments thus incentivize infrastructure investments using land-sale revenues to facilitate industrial relocation from eastern regions.”* For specific details, please refer to lines 414 to 417 of the manuscript.

7.The manuscript contains redundancy of ideas throughout the paper.

A：Thank you for your thorough review. This paper has primarily revised the cross-referenced content regarding the mechanism analysis between the theoretical framework and empirical sections to enhance conciseness while maintaining overall coherence. Notably, findings that diverge from existing studies have been relocated to the discussion section for systematic analysis. Key modifications are summarized below:

*“In order to test the role of labor transfer effect, public service supply effect and industrial structure upgrading effect in the process of the influence of local government land grant behavior on urban-rural integration development, based on equations (2), (3) and (4), the moderating effect model is used to identify the interaction effects brought by labor transfer, public service supply and industrial structure upgrading, and the regression results are shown in Table 11. The results from regression (1) in Table 11 indicate that the interaction term between labor mobility and land price competition is significantly positive, and the coefficient of land price competition itself is also significantly positive. This suggests that labor mobility strengthens the positive effect of land price competition on urban-rural integrated development, thereby validating Hypothesis H2. The results from regression (2) in Table 11 show that the interaction term between public service provision and land price competition is not statistically significant, which contradicts Hypothesis H3. The results from regression (3) in Table 11 reveal that the interaction terms between industrial structure upgrading and both land price competition and land fiscal dependence are significantly positive, confirming Hypothesis H4.”* For specific details, please refer to lines 535 to 546 of the manuscript.

*“Compared to prior studies, this paper draws extensively on key insights from the literature regarding the relationship between land markets—such as land resource misallocation [10] and factor mobility [11]—and urban-rural integration. It also references empirical methods for measuring urban-rural integration levels, land marketization, and econometric model construction [51], while extending the theoretical framework on how land market reforms shape urban-rural dynamics [24]. Our findings confirm that land price competition positively contributes to urban-rural integration, whereas fiscal dependence on land exerts a negative effect. However, some scholars argue that land finance serves as a crucial engine for regional economic development [59]. Thus, dismissing its role entirely would be unbalanced; instead, efforts should focus on diversifying local government revenue sources to gradually reduce reliance on land finance and achieve sustainable urban-rural integration. Notably, this study exhibits significant distinctions from existing research in the following aspects: First, regarding research perspective, prior literature predominantly focuses on the impact of land markets per se or land resource allocation efficiency on urban-rural integration [29]. In contrast, this paper centers on local governments' land conveyance behaviors, internalizing them into two dimensions—land price competition and fiscal dependency on land revenue—thereby offering deeper insights into the mechanisms through which local governments' land transactions influence urban-rural integration. Second, existing studies posit that high-quality public service provision fosters a conducive environment for enterprises and investors, facilitating the entry and agglomeration of external firms and thereby promoting regional industrial upgrading and economic development [60]. However, our analysis reveals that public service provision does not exhibit a significant positive moderating effect in the relationship between local governments’ land conveyance behaviors and urban-rural integration. A plausible explanation lies in the fact that while local governments’ low-price industrial land transfers may attract more enterprises and capital, stimulating economic growth and employment opportunities, they simultaneously necessitate increased public service investments (e.g., infrastructure) to sustain such development. Yet, the public services provided may fail to adequately support the ancillary needs of enterprises attracted by underpriced industrial land, thereby diminishing the anticipated reinforcing effect of public services on urban-rural integration under conditions of land price competition. Conversely, the interaction term between public service provision and fiscal dependency on land revenue shows a significantly positive coefficient. This suggests that enhancing public service levels—such as infrastructure and social security investments—can facilitate cross-regional flows of labor and other resources. Such improvements effectively mitigate adverse effects stemming from fiscal reliance on land revenue (e.g., soaring housing prices, industrial rigidity, and homogenization), thereby weakening its negative impact on urban-rural integration.”* For specific details, please refer to lines 572 to 601 of the manuscript.

8.The title of the manuscript requires modification.

A：Thank you for your suggestions. The paper's title has been revised to:

*“Research on the Impact and Mechanism of Local Governments' Land Conveyance Behavior on Urban-Rural Integrated Development——Empirical Evidence from 281 Prefecture-Level Cities in China”*

Once again, thank you very much for your comments and suggestions! Those comments are all valuable and very helpful for revising and improving our paper.

Best Wishes!
