# Supplementary material for: Research on the impact and mechanism of local governments’ land conveyance behavior on urban-rural integrated development—Empirical evidence from 281 prefecture-level cities in China
Source: PLoS One. 2025 Jul 7;20(7):e0327433. doi: 10.1371/journal.pone.0327433 (PMC12233302; doi:10.1371/journal.pone.0327433)
Supplement: Attachment — Submitted filename: Response_to_Reviewers_auresp_3.docx [file pone.0327433.s005.docx]

**Manuscript ID:**PONE-D-24-33248

**Title:**  Research on the Impact and Mechanism of Local Government Land Transfer Behavior on Urban Rural Integration Development——Perspectives based on land price competition and land finance dependence

**Authors name:**Long Zeng , Bin Peng' * and Jin Xie

**Response to Reviewers**

Dear Editor and Reviewers,

Thanks very much for taking your time to review this manuscript. we really appreciate all your comments and suggestions! Below, we provide a detailed response to each comment raised by the reviewers and outline the revisions made accordingly. In addition, the modifications we have made have been highlighted in red font in the re-submitted files.

**Reply to the comments of Reviewer 4**

1.The article deals with an interesting topic. However, according to the template, a literature review should be provided separately.

A：Thank you very much for your careful review. Based on your suggestion, this article has separated the literature review content from the current article, and the specific abstract is as follows:

*“Literature Review*

*Under the urban-rural dual structure, cities typically offer more employment opportunities and higher wages, which incentivize the migration of labor and other production factors from rural to urban areas. However, with the evolving development environment between urban and rural regions, a reverse trend has emerged, where production factors, including labor, are increasingly flowing back from cities to rural areas. Urban-rural integrated development is widely regarded as a process that facilitates the rational flow and optimal allocation of production factors between urban and rural areas, thereby promoting economic integration and interactive growth. Existing literature has explored the conceptual framework and practical challenges of urban-rural integration from a theoretical perspective [5], while also identifying its driving mechanisms and pathways [6]. Empirical studies have further measured the level of urban-rural integration and examined its influencing factors from various dimensions [7], including economic growth [8], economic agglomeration [9], misallocation of urban-rural production factors [10], factor mobility [11], water quality improvement measures [12], new industrial cooperation models [13], and infrastructure development [14]. These studies often adopt analytical units at the provincial or prefecture-city level [15].*

*The academic community has deeply explored the relationship between land sales and urban-rural integration development. As a resource element and spatial carrier of urban and rural economic development, land has become a crucial force in the evolution of China's land system reform through market mechanisms [16]. Against the backdrop of a dual urban-rural land resource allocation system [17], urban-biased land acquisition methods [18], and government competition [19], land element allocation strategies and reforms have become important tools for local governments to develop regional economies [20-21].Overall, research on the relationship between land sales and urban-rural integration development can be divided into two main categories: Indirect Impact Studies: These studies examine how local government land sale practices indirectly influence urban-rural integration development by affecting related areas such as regional industrial structure upgrading [22], economic growth [23], urbanization development [24], urban-rural income gaps [25], Agricultural efficiency [26], sustainable development [27], and public services [28]. Direct Impact and Reform Path Studies: These studies explore the mechanisms [29-30], historical evolution, mechanism design [31], and institutional innovations [32] through which the Chinese land market impacts urban-rural integration development. On this basis, scholars further analyze the effects of land resource misallocation [11], land resource allocation efficiency [33], land use rates, and land transformation [34] on urban-rural integration development.*

*In summary, existing research has provided a significant theoretical and empirical foundation for this study. Existing literature predominantly focuses on the impacts of land markets, land resource allocation efficiency, and misallocation on urban-rural integration, while paying relatively little attention to the effects of local governments' land transfer behaviors on urban-rural integration. Furthermore, previous studies have not systematically conceptualized local governments' land transfer behaviors into the two dimensions of land price competition and land fiscal dependence to explore their impacts and heterogeneity concerning urban-rural integration. Additionally, the mechanisms through which local governments' land transfer behaviors influence urban-rural integration remain largely unexplored. The potential marginal contributions of this study are as follows: First, this paper is the first to systematically elucidate the impact mechanisms of local governments' land transfer behaviors on urban-rural integration, filling a gap in the existing literature. By constructing a unified theoretical framework, this study deeply examines how land transfer behaviors influence urban-rural integration through mechanisms such as labor migration, public service provision, and industrial structure upgrading. This provides a novel perspective for understanding the relationship between land transfer behaviors and urban-rural integration. Second, this study offers theoretical support and practical guidance for optimizing land transfer policies by local governments. In particular, in the context of promoting urban-rural integration, the findings of this paper help local governments better understand the multidimensional impacts of land transfer behaviors, enabling them to formulate more scientific land transfer and fiscal policies. Furthermore, the policy recommendations provided in this study can help governments rationally adjust their land transfer practices to promote labor migration, enhance public service provision, and facilitate industrial structure upgrading, thereby fostering urban-rural integration, reducing the urban-rural gap, and achieving coordinated economic and social development.”*

For specific details, please refer to lines 93 to 141 of the manuscript.

2.The article addresses the issue in medias res without a theoretical background. It would be appropriate to supplement the theoretical background of the problem being addressed, how the given topic is being addressed abroad.

A：Thank you very much for your suggestion. This article has made modifications to the introduction section and supplemented the theoretical background of the research content and empirical evidence on how foreign countries handle it. The specific summary is as follows：

*“The integrated development of urban and rural areas serves as a fundamental pathway for China's comprehensive rural revitalization and a crucial measure to dismantle the urban-rural duality and achieve common prosperity. As explicitly stated in the report of the 20th National Congress of the Communist Party of China, efforts should be made to promote urban-rural integration and coordinated regional development, facilitate the circulation of the national economy, and establish a new development dynamic between urban and rural areas. Consequently, how to achieve integrated urban-rural development has become a significant proposition in China's current phase of new development. From a theoretical perspective, research on urban-rural integrated development is rooted in theories of urban-rural relations, spatial economics, and institutional economics. Lewis's dual-sector model first revealed the economic structure of urban-rural segmentation in developing countries, highlighting the coexistence of traditional agricultural sectors and modern industrial sectors. The Todaro model further analyzed the economic motivations behind rural-urban migration, emphasizing the role of expected income differentials in labor mobility. Meanwhile, new economic geography explains, from a spatial perspective, how industrial agglomeration and dispersion shape urban-rural relations. These theories provide an essential analytical framework for China's pursuit of urban-rural integrated development.*

*Internationally, developed countries have cultivated relatively mature models for advancing urban-rural integration. The United States, for example, introduced the Smart Growth Program to establish metropolitan governance mechanisms, enhance infrastructure interconnectivity, and foster coordinated urban-rural planning. Germany implemented its "Urban-Rural Equalization" strategy, leveraging spatial planning laws and fiscal equalization to safeguard rural development rights. Japan successfully elevated rural industrial competitiveness through municipal mergers and the "One Village, One Product" initiative, while South Korea’s New Village Movement systematically upgraded rural production and living standards through government-guided, community-driven participation. These cases collectively underscore that land institutional reform, equitable public service allocation, and industrial synergy constitute pivotal pathways to achieving urban-rural integration. From the process of promoting urbanization and rural revitalization in China, it is evident that adjusting the irrational allocation of resources can achieve balanced, shared, and integrated development between urban and rural areas [1]. As a spatial carrier and production factor of urban and rural regional systems and production activities, land's use and allocation changes impact the circulation of resources between urban and rural areas, playing a crucial role in transforming the urban-rural structure and driving economic growth [2].Given that the allocation of land elements in China is primarily government-led, local governments not only provide the foundational conditions for regional economic development by selling industrial land at low prices to attract foreign investment and industrial agglomeration but also generate financial resources for regional economic development by selling commercial and residential land at high prices [3]. This aids in promoting urban-rural integration development. However, the long-standing urban-biased development approach in China has led to issues such as land segmentation, separation of people and land, and urban-rural division. These issues can result in irrational land sale practices that hinder urban-rural integration development [4].Thus, how do local government land sale practices impact urban-rural integration development? Addressing this question not only clarifies the mechanisms by which local government land sale practices influence urban-rural integration development but also provides theoretical and practical bases for local governments to use land resource allocation to promote urban-rural integration development. ”*

For specific details, please refer to lines 55 to 92 of the manuscript.

3.The article is appropriately processed from a methodological point of view, but the limits of the research are missing in the conclusion.

A：Thank you very much for your careful review. Although the limitations of the study were discussed in the discussion section, based on your suggestion, this article has added the conclusion of the study and a summary of the limitations in the conclusion section. The specific summary is as follows:

*“This study employs panel data from 281 prefecture-level and above cities in China, utilizing bidirectional fixed-effects models and moderated mediation models to analyze local governments' land conveyance practices. These practices are categorized into two dimensions—low-priced industrial land transfers and high-priced commercial/residential land transfers—and examined within a unified theoretical framework. The research systematically establishes the causal relationship between local governments' land conveyance strategies and urban-rural integrated development, while empirically analyzing their effects and underlying mechanisms. However, several limitations remain: (1) potential minor discrepancies between the constructed urban-rural integration index system and actual integration levels; (2) possible omission of other control variables that may influence urban-rural development; and (3) insufficient examination of whether moderating variables indirectly affect urban-rural integration through mediating variables.”*

For specific details, please refer to lines 633 to 642 of the manuscript.

Once again, thank you very much for your comments and suggestions! Those comments are all valuable and very helpful for revising and improving our paper.

Best Wishes!
